# Negative Contrast Esophagography Does Not Exclude Dehiscence After Boerhaave Repair: Complementary Low-Insufflation Carbon Dioxide Endoscopy

**DOI:** 10.3390/diagnostics16111670

**Published:** 2026-05-28

**Authors:** Hee Suk Jung, Gong Min Rim, Young Mog Shim, Kwan Wook Kim

**Affiliations:** Department of Thoracic and Cardiovascular Surgery, CHA Bundang Medical Center, CHA University, 59 Yatap-ro, Bundang-gu, Seongnam-si 13496, Republic of Korea; hsjung80@cha.ac.kr (H.S.J.);

**Keywords:** Boerhaave syndrome, esophageal perforation, contrast esophagography, endoscopy, carbon dioxide, dehiscence

## Abstract

After repair of Boerhaave syndrome, assessment of repair integrity is important because occult dehiscence may progress to mediastinitis or pleural sepsis. Water-soluble contrast esophagography is widely used before resuming oral intake, but absence of extravasation does not necessarily exclude a small or contained defect. We report a 59-year-old man with post-emetic distal esophageal rupture treated by emergency primary repair with pleural-flap buttress and drainage. Although clinically stable, contrast esophagography on postoperative day (POD) 7 showed no extravasation. Because feeding decisions required greater structural confidence, low-insufflation carbon dioxide esophagogastroduodenoscopy was performed on POD 9 and revealed focal suture-line dehiscence. Oral intake was deferred and conservative management continued. A regular diet was started only after POD 28 endoscopy showed marked interval healing; 3-month follow-up confirmed complete mucosal healing. Contrast esophagography remains the standard postoperative assessment; nevertheless, this hypothesis-generating case suggests that, in carefully selected, clinically stable patients with high-risk anatomic features, low-insufflation carbon dioxide endoscopy may provide complementary structural information when feeding decisions remain uncertain after a negative contrast study.

**Figure 1:**
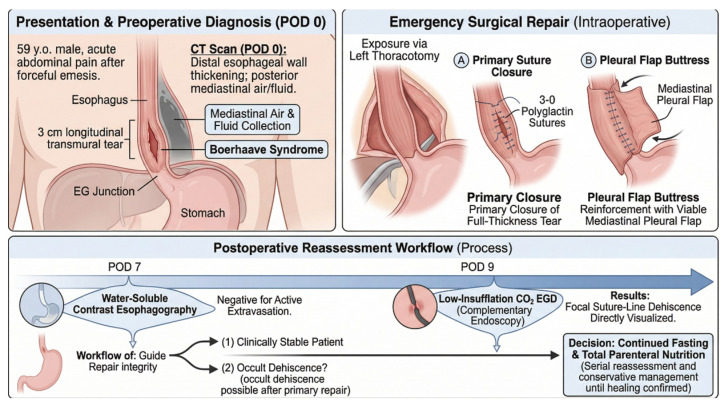
Clinical presentation, emergency surgical repair, and postoperative reassessment workflow in Boerhaave syndrome. A 59-year-old man presented with acute epigastric and right upper-quadrant pain after forceful emesis. The patient experienced sudden forceful emesis at approximately 15:15 on the day prior to admission, followed by acute epigastric and right upper-quadrant pain. He presented to the emergency department at approximately 15:00 the following day, that is, approximately 24 h after symptom onset. Laboratory tests revealed leukocytosis with neutrophilia (white blood cell count, 14.45 × 10^3^/μL; neutrophils, 85.6%), with elevated C-reactive protein (CRP, 18.49 mg/dL) and a body temperature of 38.4 °C on arrival (see Figure 2A). Contrast-enhanced abdominopelvic computed tomography demonstrated distal esophageal transmural perforation with posterior mediastinal air and fluid, consistent with Boerhaave syndrome [1,2]. Emergency surgery was initiated at approximately 16:30 on the day of admission, approximately 1.5 h after emergency department arrival and approximately 25 h after symptom onset. Emergency surgery revealed a 3 cm longitudinal full-thickness tear just proximal to the esophagogastric junction. The defect was closed primarily with interrupted 3-0 polyglactin (Vicryl; Ethicon, Inc., Raritan, NJ, USA) sutures (**A**) and buttressed with a mediastinal pleural flap (**B**). The 3 cm full-thickness tear at this distal location was considered a high-risk site because of its proximity to the esophagogastric junction and the elevated transmural pressures generated during deglutition and reflux. Despite a presentation that exceeded the classical 24 h threshold often used for primary surgical repair, primary repair with pleural-flap buttressing was selected because the patient remained hemodynamically stable, the tissue at the rupture margins was viable at intraoperative inspection, and adequate mediastinal and pleural source control could be achieved in a single procedure. Primary endoscopic management alone (e.g., stent placement or endoluminal closure) was not selected because of the size and full-thickness nature of the defect, the need for mediastinal debridement and pleural drainage for source control, the recognized high migration rate of esophageal stents placed across the esophagogastric junction, and the limited evidence supporting endoluminal closure as a standalone modality in the setting of established transmural contamination. Postoperatively, the patient was managed with fasting, drainage, and broad-spectrum intravenous antibiotics for 7 days. He remained hemodynamically stable without febrile episodes, and inflammatory markers progressively declined on serial testing (white blood cell count 15.3 → 8.5 × 10^3^/μL and CRP 15.2 → 5.6 mg/dL from POD 1 to POD 7; Figure 2A). Thoracic drainage was consistently serosanguineous without salivary, enteric, bilious, or food-particle features, and decreased from approximately 360 mL on POD 1 to 230 mL on POD 7 (detailed serial data are provided in Figure 2B). The postoperative workflow is summarized from POD 7 to POD 9, highlighting the discrepancy between negative water-soluble contrast esophagography and subsequent endoscopic detection of focal dehiscence.

**Figure 2:**
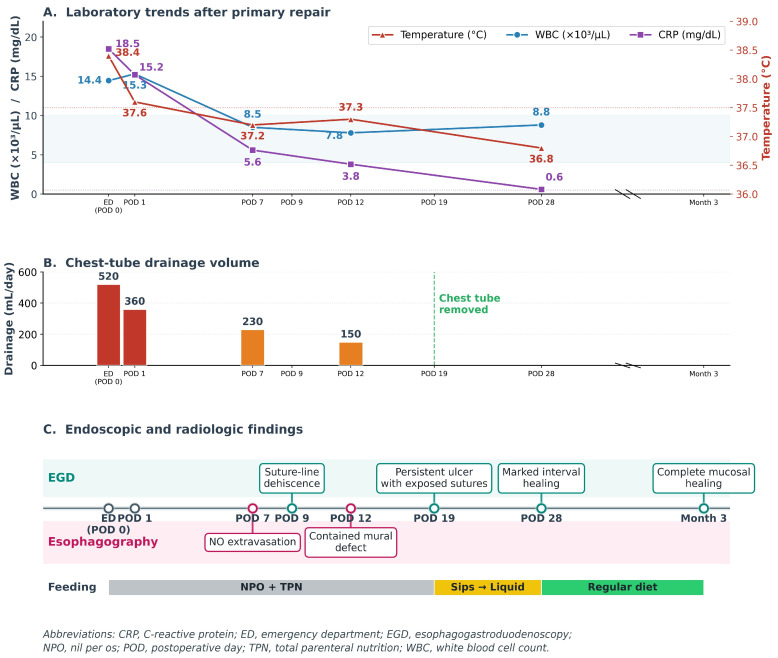
Postoperative timeline of biochemical, radiologic, and endoscopic findings after primary repair of Boerhaave syndrome. (**A**) Trends in body temperature, white blood cell (WBC) count, and C-reactive protein (CRP) from emergency department admission (POD 0) through POD 28; shaded bands indicate the reference ranges. WBC normalized and body temperature defervesced by POD 7, with a progressive decline in CRP through POD 28. (**B**) Daily chest-tube drainage volume; drainage was consistently serosanguineous and decreased progressively, and the chest tube was removed shortly after cautious resumption of oral intake. (**C**) Integrated chronological timeline mapping endoscopic (EGD) findings and water-soluble contrast esophagography findings against feeding status (NPO + TPN → sips/liquid → regular diet) across the full postoperative course. This figure consolidates the clinical course in a single visual reference, allowing rapid scanning of the discordance between POD 7 contrast esophagography (no extravasation) and POD 9 endoscopy (focal suture-line dehiscence), as well as the subsequent contained mural defect on POD 12, persistent ulcer with exposed Vicryl sutures on POD 19, marked interval healing on POD 28, and complete mucosal healing at the 3-month follow-up.

**Figure 3:**
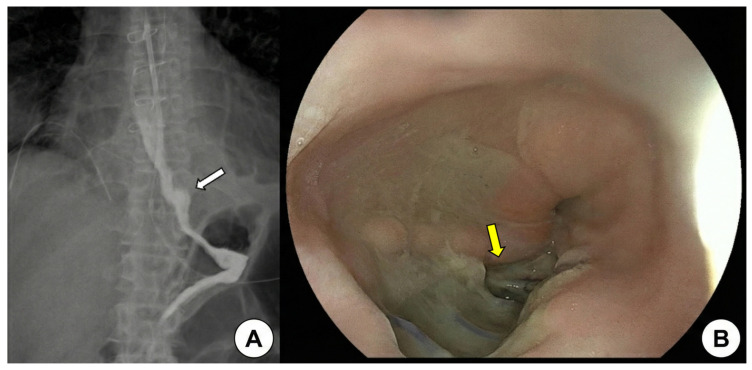
Discordant early postoperative assessments after primary repair of Boerhaave syndrome (POD 7–9). (**A**) Water-soluble contrast esophagography (Gastrografin; Bayer AG, Berlin, Germany) on POD 7 shows prompt passage of contrast into the stomach without free extravasation (arrow). Water-soluble contrast esophagography is widely used because it is noninvasive and highly specific for active extraluminal contrast escape, but false-negative examinations may occur when defects are small, intermittently leaking, or functionally contained, or when the examination is insufficiently provocative [1,3]. In addition, a defect that is small or intermittently leaking may fail to opacify during a single contrast examination, and surrounding inflammatory tissue together with the pleural-flap buttress may functionally seal the defect under the low transmural pressures generated during contrast swallow. Therefore, the absence of free contrast extravasation indicates the absence of an active free leak at the time of examination, but does not necessarily prove complete structural healing of the mucosa or suture line. Prior studies in postoperative esophageal surgery have shown that some leaks are not detected on the initial contrast study [4,5]. When concern persists despite a negative contrast study, computed tomography may provide complementary information regarding extraesophageal air, fluid, or collections [1,6]. (**B**) Because oral feeding decisions required more confident structural reassessment, low-insufflation CO_2_ EGD was performed on POD 9 with strict minimization of luminal distension and demonstrated focal suture-line dehiscence at the repair site. The decision to perform EGD was individualized rather than protocol-driven. The patient remained hemodynamically stable and afebrile, with progressively improving inflammatory markers and decreasing, non-contaminated thoracic drainage (Figure 2); the original 3 cm full-thickness tear at the high-risk distal location and the impending decision regarding oral intake led to a multidisciplinary decision to obtain direct endoscopic visualization of the repair site before reintroducing enteral feeding. The procedure was performed by a senior interventional endoscopist using a standard diagnostic gastroscope, in close real-time communication with the surgical team. CO_2_ insufflation was applied with strict minimization of luminal distension throughout the procedure. Forceful insufflation, repeated mechanical contact with the repair site, and unnecessary advancement or torqueing across the repaired segment were avoided. The examination was intended for careful visual inspection only; no therapeutic manipulation or provocative leak testing was performed. The endoscopic finding is consistently termed “focal suture-line dehiscence” because EGD directly visualized mucosal and suture-line disruption at the repair site, in distinction to the radiologic concept of a “contained defect” used below. Importantly, early postoperative EGD should not be considered in patients who are hemodynamically unstable, septic, who have undrained mediastinal or pleural contamination, radiologically evident free extravasation, or a suspected large uncontained fistula. More specifically, although the patient was clinically stable and contrast esophagography was negative, several anatomic and procedural features raised residual concern that a small or intermittently leaking defect could remain below the detection threshold of a single contrast study: (i) the 3 cm full-thickness nature of the original tear; (ii) its location immediately proximal to the esophagogastric junction, where transmural pressures during deglutition and gastroesophageal reflux are highest; (iii) the use of a pleural-flap buttress that could functionally seal a small defect under the low transmural pressures of a contrast swallow; and (iv) the clinical consequence that premature oral intake at this level could rapidly convert a contained dehiscence into overt mediastinitis. The combination of these features, rather than any single new clinical sign, constituted the specific suspicion that prompted endoscopic reassessment. The risk–benefit balance was explicitly discussed by the surgical and endoscopic teams before the procedure. The principal anticipated risks were mechanical extension of the suture-line defect by luminal distension, displacement of the pleural-flap buttress, and aggravation of any subclinical mediastinal contamination. These were judged acceptable because (i) the patient was hemodynamically stable, afebrile, with declining inflammatory markers and non-contaminated thoracic drainage, providing a controlled physiologic window; (ii) CO_2_ rather than room-air insufflation was used, given the rapid mucosal absorption of CO_2_ and its more favorable safety profile if any minor extraluminal escape were to occur; (iii) the examination was strictly diagnostic, with no provocative leak testing, biopsy, or therapeutic manipulation planned; and (iv) the alternative of empirically resuming oral intake on the basis of a negative contrast study alone was considered higher-risk given the anatomic features outlined above. Oral intake was therefore withheld, and fasting with total parenteral nutrition was continued. Although concerns have historically existed that endoscopy may worsen a defect by insufflation, current practice emphasizes risk reduction with CO_2_ insufflation and strict minimization of luminal distension when perforation is suspected [7]. Early postoperative endoscopy after esophageal surgery has also been reported to be safe and clinically informative in selected settings [8]. More recently, a large propensity score-matched cohort of 941 patients undergoing minimally invasive esophagectomy demonstrated that even ultra-early postoperative endoscopy performed within 24 h did not increase postoperative adverse events or in-hospital mortality, and was independently associated with a lower incidence of anastomotic leak (odds ratio 0.476, 95% confidence interval 0.315–0.718, *p* < 0.001) and shorter hospital stay compared with endoscopy performed between 24 and 72 h [9]. Although these data derive from anastomosis after esophagectomy rather than primary repair of a transmural rupture, they support the broader principle that, in appropriately selected patients, a carefully performed postoperative esophageal endoscopy can be safe and may meaningfully inform clinical decision-making. The yellow arrow in (**B**) indicates the focal suture-line dehiscence at the repair site.

**Figure 4:**
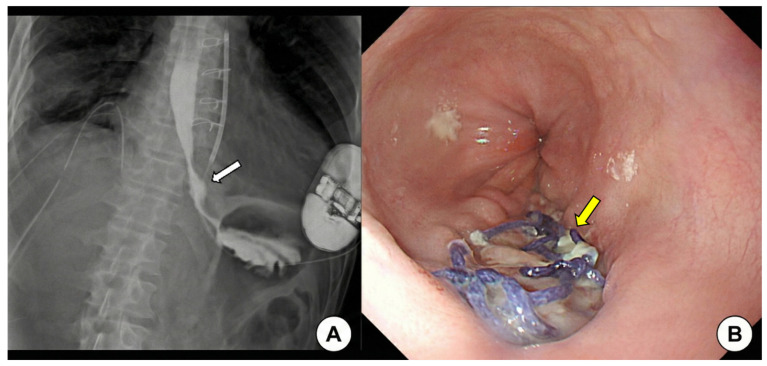
Serial findings suggesting a contained defect during conservative management (POD 12–19). (**A**) Repeat water-soluble contrast esophagography on POD 12 demonstrates an approximately 2.5 cm ulcerated mural defect in the distal esophagus without free extravasation (arrow), consistent with a contained defect rather than complete healing or an active free leak—distinct from the focal suture-line dehiscence directly visualized endoscopically on POD 9. The absence of free extraluminal contrast indicates functional containment under the low transmural pressures of a contrast swallow but does not by itself confirm structural mucosal healing [1,3]. For the purposes of this report, a contained defect is operationally defined as a postoperative esophageal wall discontinuity in which (i) contrast esophagography demonstrates a localized intramural or paraluminal collection or ulcerated mural irregularity without free extraluminal contrast extravasation; (ii) cross-sectional imaging shows no expanding extraluminal air or fluid collection beyond the immediate repair site; and (iii) the patient remains hemodynamically stable without systemic signs of uncontrolled sepsis. This radiologic–clinical construct is distinct from focal suture-line dehiscence, which in this report refers to direct endoscopic visualization of mucosal and suture-line disruption at the repair site irrespective of contrast behavior. In a clinically stable patient without systemic sepsis, such findings may support continued conservative management with close monitoring and repeat reassessment, whereas uncontained leakage typically warrants prompt escalation [1,2]. (**B**) Follow-up low-insufflation CO_2_ EGD on POD 19 shows a persistent ulcer at the repair site with exposed suture material. This procedure was again performed by the same senior interventional endoscopist using a standard diagnostic gastroscope under low-insufflation CO_2_ technique with strict minimization of luminal distension and no provocative testing. Because the patient remained hemodynamically stable and showed no evidence of systemic deterioration, nonoperative management was continued. After this reassessment, oral intake was cautiously reintroduced with small amounts and was tolerated without clinical worsening. The chest tube was removed 3 days after resumption of oral intake. The yellow arrow in (**B**) indicates exposed Vicryl suture loops at the repair site.

**Figure 5:**
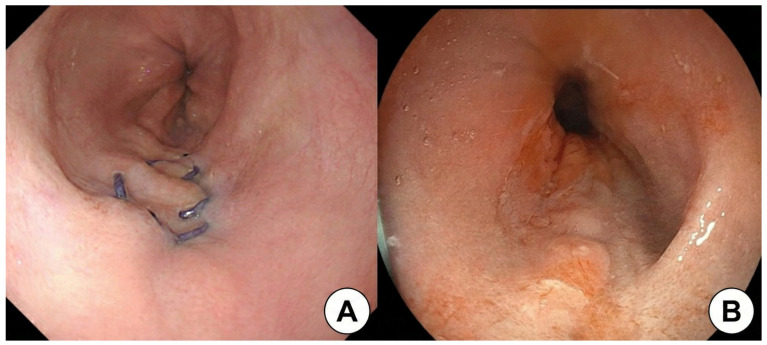
Serial endoscopic documentation of healing after primary repair of Boerhaave syndrome. (**A**) Low-insufflation CO_2_ EGD on POD 28 shows marked interval healing at the repair site without endoscopic evidence of an open transmural defect; residual sutures are partially covered by regenerated mucosa. The POD 28 examination was performed by the same senior interventional endoscopist using the same low-insufflation CO_2_ technique, with careful avoidance of forceful luminal distension or provocative leak testing. After this examination, the patient was advanced to a regular diet, which was tolerated without dysphagia or systemic deterioration. (**B**) Follow-up endoscopy at approximately 3 months confirms complete mucosal healing without evidence of recurrent leakage or infectious complications. It should be noted that current international guidance on esophageal emergencies, including the World Society of Emergency Surgery guidelines [1], does not provide a standardized postoperative surveillance algorithm specifying which patients require contrast esophagography alone, which require cross-sectional imaging, and which may benefit from direct endoscopic reassessment before oral intake is resumed. Existing recommendations on contrast esophagography are largely extrapolated from the anastomotic-leak literature after esophagectomy [4,5], where the underlying tissue, repair technique, and luminal pressures differ from those after primary repair of a transmural rupture. This guideline gap leaves the timing, modality, and threshold for postoperative reassessment to local practice. The present case therefore suggests a potential complementary workflow in which a negative postoperative contrast esophagography in a clinically stable patient with high-risk anatomic features (e.g., a large defect or one located at or near the esophagogastric junction) should not by itself trigger immediate resumption of oral intake. In such selected scenarios, low-insufflation CO_2_ EGD performed by an experienced endoscopist in close coordination with the surgical team may provide direct structural confirmation before the feeding decision, whereas in low-risk patients with concordant clinical and radiologic evidence of healing, contrast esophagography alone may remain sufficient. Limitations of the present report include its single-patient, single-center nature, performance by a single experienced operator, the absence of recorded numeric insufflation pressure or flow settings, and the absence of comparative data; prospective, multicenter studies will be required to formally evaluate the role of selective low-insufflation CO_2_ EGD within a risk-stratified algorithm. This case is hypothesis-generating. It illustrates that absence of extravasation on postoperative contrast esophagography should not automatically be interpreted as absence of dehiscence. Contrast esophagography remains the standard postoperative assessment after esophageal repair; however, in selected clinically stable patients in whom feeding decisions depend on structural reassessment, carefully performed low-insufflation CO_2_ endoscopy may serve as an auxiliary clinical tool providing complementary information when performed by experienced operators under strict safety conditions [1,3,4,5,6,7,8,9].

## Data Availability

The original contributions presented in this study are included in the article. Further inquiries can be directed to the corresponding author.
